# SARS-CoV-2 analysis on environmental surfaces collected in an intensive care unit: keeping Ernest Shackleton’s spirit

**DOI:** 10.1186/s40635-020-00349-5

**Published:** 2020-11-23

**Authors:** Dolores Escudero, José Antonio Boga, Javier Fernández, Lorena Forcelledo, Salvador Balboa, Rodrigo Albillos, Iván Astola, Emilio García-Prieto, Marta Elena Álvarez-Argüelles, Gabriel Martín, Josu Jiménez, Fernando Vázquez

**Affiliations:** 1 Servicio de Medicina Intensiva, Hospital Universitario Central de Asturias (HUCA), Oviedo, Spain; 2 Grupo de Investigación Microbiología Traslacional, Instituto de Investigación Sanitaria del Principado de Asturias (ISPA), Oviedo, Spain; 3 Servicio de Microbiología, Hospital Universitario Central de Asturias (HUCA), Oviedo, Spain; 4 Grupo de Investigación Traslacional en el Paciente Crítico, Instituto de Investigación Sanitaria del Principado de Asturias (ISPA), Oviedo, Spain; 5 CIBER-Enfermedades Respiratorias, Instituto de Salud Carlos III, Madrid, Spain; 6 Departamento de Medicina, Universidad de Oviedo, Oviedo, Spain; 7 Servicio de Ingeniería y Mantenimiento, Hospital Universitario Central de Asturias (HUCA), Oviedo, Spain; 8 Fundación de Investigación Oftalmológica, Instituto Oftalmológico Fernández-Vega, Oviedo, Spain

**Keywords:** SARS-CoV-2, COVID-19, ICU, ICU environmental contamination

## Abstract

**Background:**

Intensive care unit workers are at high risk of acquiring COVID-19 infection, especially when performing invasive techniques and certain procedures that generate aerosols (< 5 μm). Therefore, one of the objectives of the health systems should implement safety practices to minimize the risk of contagion among these health professionals. Monitoring environmental contamination of SARS-CoV-2 may help to determine the potential of the environment as a transmission medium in an area highly exposed to SARS-CoV-2, such as an intensive care unit. The objective of the study was to analyze the environmental contamination by SARS-CoV-2 on surfaces collected in an intensive care unit, which is dedicated exclusively to the care of patients with COVID-19 and equipped with negative pressure of – 10 Pa and an air change rate of 20 cycles per hour. Furthermore, all ICU workers were tested for COVID-19 by quantitative RT-PCR and ELISA methods.

**Results:**

A total of 102 samples (72 collected with pre-moistened swabs used for collection of nasopharyngeal exudates and 30 with moistened wipes used in the environmental microbiological control of the food industry) were obtained from ventilators, monitors, perfusion pumps, bed rails, lab benches, containers of personal protective equipment, computer keyboards and mice, telephones, workers’ shoes, floor, and other areas of close contact with COVID-19 patients and healthcare professionals who cared for them. The analysis by quantitative RT-PCR showed no detection of SARS-CoV-2 genome in environmental samples collected by any of the two methods described. Furthermore, none of the 237 ICU workers was infected by the virus.

**Conclusions:**

Presence of SARS-CoV-2 on the ICU surfaces could not be determined supporting that a strict cleaning protocol with sodium hypochlorite, a high air change rate, and a negative pressure in the ICU are effective in preventing environmental contamination. These facts together with the protection measures used could also explain the absence of contagion among staff inside ICUs.

## Background

In December 2019, a new betacoronavirus causing pneumonia and acute respiratory distress syndrome was detected in China. The virus and the disease were called SARS-CoV-2 and Coronavirus Disease (COVID)-19, respectively [1–3]. In a few months, it spread rapidly throughout the world being officially declared as a pandemic by the World Health Organization (WHO) on 11 March 2020 [4].

The main modes of transmission of the virus is through respiratory droplets (> 5 μm), aerosols (< 5 μm), and fomites contaminated with respiratory secretions [5, 6]. Intensive care unit (ICU) workers are at high risk when performing invasive techniques and certain procedures that generate aerosols while working in an environment highly exposed to SARS-CoV-2. Therefore, one of the objectives of the health systems should implement safety practices to minimize the risk of contagion among health professionals.

The aim of this study is to analyze the environmental contamination by SARS-CoV-2 on surfaces of an ICU dedicated to treating patients with COVID-19.

## Methods

**Fig. 1:**
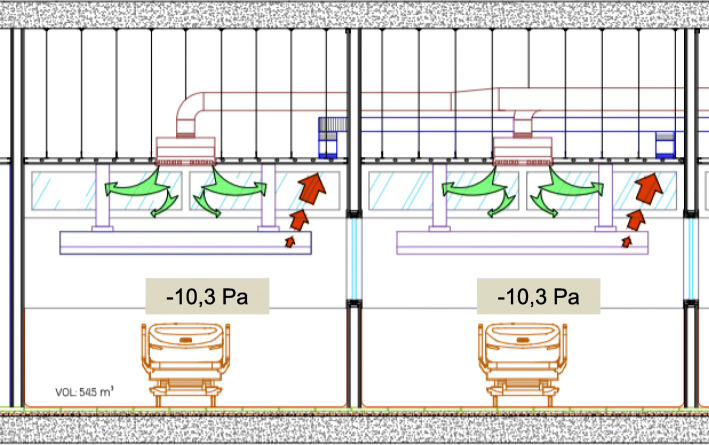
Representation of the directions of the air flow in ICU rooms. Fresh air (green arrows) and dirty air (red arrows)

**Fig. 2:**
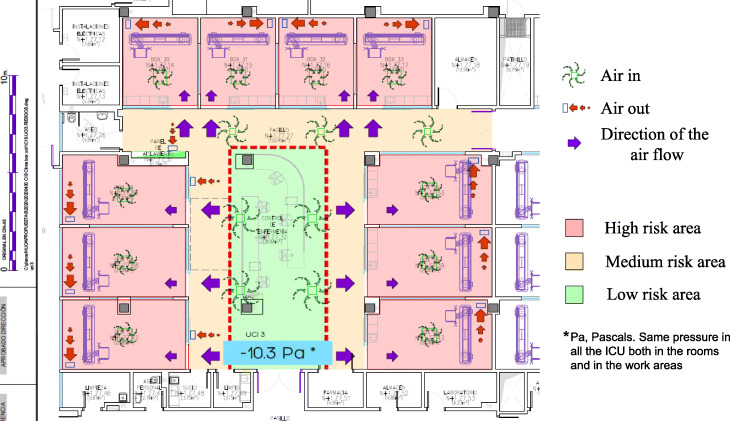
Map showing the high, medium, and low risk areas, as well as directions of the air flows of the intensive care unit. The value of the negative pressure is indicated

From February 29th to June 5th, a total of 86 patients with COVID-19 were admitted in our hospital (62 in a multipurpose ICU and 24 in a cardiac ICU managed by specialists in intensive medicine). The multipurpose ICU had 44 beds distributed in 5 units (4 units with 10 beds and 1 unit with 4 beds, all boxes are independent with a surface of 22 m^2^). During the studied period, the acute phase of the pandemic, all ICU beds were exclusively used to treat patients diagnosed with COVID-19. All the patients had a high level of severity; most of them required mechanical ventilation, and some presented failure of other organs such as kidney failure with hemofiltration. The space within the ICU was divided into three areas of risk exposure (high, medium, and low). Inside the boxes (high risk area), the professionals worked with personal protective equipment (PPE). A security perimeter, 2 m from the entrance of the isolation rooms, was marked on the ground with a red stripe (medium risk area). In this area, professionals had to use a surgical mask. The central area of the unit, which is a space intended for administrative work with electronic medical records, was considered as a low risk area and work without a mask was allowed. All the ICU units were equipped with negative pressure of – 10 Pa and an air flow circuit with circulation from the central area to the boxes with an air change rate of 20 cycles per hour (Figs. 1 and 2). The mean temperature was 22 °C (range 22–24 °C) and the humidity was 50% (range 45–55%). The ICU was cleaned with detergent and 0.05% sodium hypochlorite in the morning and afternoon shift. Some surfaces such as the pagers or the EPI face masks were disinfected with 70% ethanol following the recommendations of the ECDC [7].

From 16 to 27 April, 102 environmental samples were collected in the three risk areas at different times in the morning and afternoon using two methods: (1) the first 72 samples were collected by pre-moistened sterile swabs (Copan Liquid Amies Elution Swab, Copan Diagnostics, Murrieta, CA), which are used to collect nasopharyngeal exudates and 2) the next 30 samples were collected by moistened wipes 23 × 23 cm (WIPES CHI 100N, Biomerieux, Marcy-l’Etoile, France), which are commonly used in the collection of environmental samples for microbiological control in the food industry, detecting pathogens such as *Listeria monocytogenes* or *Salmonella* spp. A larger surface can be covered with this method. All samples were immediately sent to the laboratory and processed to SARS-CoV-2 detection.

The presence of SARS-CoV-2 was analyzed by detecting viral genome using a multiple quantitative retrotranscriptase (RT)-PCR. Nucleic acids were purified by MagNa Pure 96 System (Roche, Geneva, Switzerland) from the swabs transport medium or from the eluates obtained after incubating the wipes in MEM medium (Dominique Dutscher, Brumath, France) at 37 °C for half an hour. The extracts were subjected to an amplification reaction using TaqMan Fast 1-Step Master Mix (Life technologies, Carlsbad, CA) supplemented with a mixture of primers (Thermo Fisher Scientific, Walthman, MA) and taqman MGB probes (Applied Biosystems, Foster City, CA) directed against ORF1ab and N genes (Table 1). Amplifications and subsequent analysis were carried out using the Applied Biosystems 7500 Real-time PCR System.

**Table 1:** Primers and probes used to detect SARS-CoV-2

Design	Position	Name	Sequence (5′-3′)	Gen
In house	Sense primer	CoV-2-OVI-S	ATCAAGTTAATGGTTACCCTAACATGT	
	Antisense primer	CoV-2-OVI-A	AACCTAGCTGTAAAGGTAAATTGGTACC	ORF1ab
	Probe MGB FAM	CoV-2-OVI-FAM	CCGCGAAGAAGCTA	
CDC^1^	Sense primer	2019-nCoV_N1-F	GACCCCAAAATCAGCGAAAT	
	Antisense primer	2019-nCoV_N1-R	TCTGGTTACTGCCAGTTGAATCTG	Gen N
	Probe MGB VIC	2019-nCoV_N1-P-VIC	CCGCATTACGTTTGGT^2^	

^1^[8]

^2^Probe sequence has been shortened as it is a MGB probe

## Results

A total of 72 environmental samples including floor, ventilators, perfusion pumps, monitors, bed rails, containers, computer keyboards, and mice or workers’ shoe soles were initially collected using pre-moistened sterile swabs in the three risk areas (Table 2). No SARS-CoV-2 genome was detected in any of the samples. To discard that the area of collection was small, a larger surface was analyzed by using moistened wipes. Thus, a new batch of 30 samples, whose type and number are shown in Table 3, was collected in the same risk areas using moistened wipes. Although covered surface was significantly larger, no genome of SARS-CoV-2 was detected.

**Table 2:** Environmental samples (*n* = 72) collected in the ICU by pre-moistened sterile swabs and PCR results

Sample (***n***)	PCR
Door knob (2)	nd
Chair (1)	nd
Telephone (4)	nd
Keyboard computer (8)	nd
Mouse computer (3)	nd
Sink faucet (4)	nd
Perfusion pump (3)	nd
Cart (3)	nd
Door handle (1)	nd
ICU workers’ shoe sole (13)	nd
Table (3)	nd
Bench (6)	nd
Bed, bed rail, mattress (8)	nd
Ventilator (6)	nd
Bag valve mask (2)	nd
Blood pressure cuff (2)	nd
ECG electrodes (1)	nd
Oxygen supply system (2)	nd

*nd* no detectable signal or Ct > 40

**Table 3:** Environmental samples (*n* = 30) collected in the ICU by moistened wipes and PCR results

Sample (***n***)	PCR
Door knob (1)	nd
Chair (1)	nd
Waste container (2)	nd
Sink faucet (1)	nd
Perfusion pump (3)	nd
Tracheal tube (exterior) (1)	nd
ICU workers’ shoe sole (3)	nd
Sling (1)	nd
Bench (5)	nd
Bed, bed rail (4)	nd
Ventilator (3)	nd
Ward floor (3)	nd
Mouse/keyboard computer (2)	nd

*nd* no detectable signal or Ct > 40

## Discussion

A report published in April 2020 by the European Center for Disease Control and Prevention (ECDC) highlights that the percentage of healthcare professionals infected by SARS-CoV-2 in Spain is the highest in the world with 20% of all reported cases, followed by Italy (10%), China (3.8%) and the USA (3%) [9]. According to data from the Spanish Ministry of Health, 52,643 health workers had been infected on July 9, representing more than 22% of all infections [10]. At the end of April, 237 workers from the ICU of our hospital were tested for detection of SARS-CoV-2 genome and antibodies by PCR and ELISA technique, respectively. All of them were negative suggesting a low circulation of the virus among these professionals.

Some experimental studies have reported that SARS-CoV-2 can remain viable in the air generated by aerosols for up to 3 h, and on surfaces of copper, cardboard, stainless steel, and plastic for 4, 24, 48, and 72 h respectively [11]. Previous studies have also found other coronaviruses, such as SARS and MERS, in air samples within aerosols suggesting a possible air transmission [12]. Guo et al. [13] analyzed environmental contamination in surfaces collected in several yards of a hospital in Wuhan, including an ICU with an air change rate of 16 cycles per hour (no equipment with negative pressure is reported). They found that the most contaminated surfaces were those that had frequent contact with the hands of workers and patients, such as computer mice, trash cans, sickbed handrails and door knobs. Furthermore, Guo et al. [13] found that virus was detected in 70% of the ICU floor samples and half of the medical staff’s shoe soles, suggesting a possible function of the shoes as carriers of the virus. Santarpia et al. [14] analyzed surface and aerosol samples collected in an isolation unit for asymptomatic or mildly ill patients and in a hospitalization area of the University of Nebraska Medical Center by RT-PCR for SARS-CoV2. Although the method of collection is not explained in the study, the virus was detected in a high percentage of personal objects such as mobile phones, television remote controls, personal computers or patient lenses, as well as on environmental surfaces such as bed rails, floor, vents, and medical equipment of patients with COVID-19 (pulse oximetry, nasal cannula, and incentive spirometer). Razzini K et al. [15] found SARS-CoV-2 RNA in 37 environmental samples. Viral RNA was detected in 35% and 50% of the samples obtained in the ICU and in areas considered semi-contaminated (undressing room), respectively. No viral RNA was detected in clean areas. Other authors also found environmental contamination in the air and surfaces of different hospital areas [16–18] in very different proportions (5–52% of the samples). Although Zhou et al. found viral RNA in 52% of the environmental samples in a London hospital, they could not grow the virus in Vero E6 and Caco 2 cells. This fact support that genome detection does not imply the viability and infective capacity of the virus [18]. These authors also reported a surprisingly low environmental contamination in the ICU in relation to other hospital areas [18]. Other studies also reported environmental contamination in towel bowl of the bathrooms suggesting that fecal shedding could be a potential route of transmission [19]. Although these studies showed a significant environmental contamination in areas with COVID19 patients based on detection of RNA from SARS-CoV-2, no infection among health workers was reported, which is explained by the implementation of effective protection measures [14].

In contrast to these studies, Colaneri et al. [20] did not detect SARS-CoV-2 genome in environmental samples collected using moistened swabs in a hospital in Northern Italy. Such as the authors state, the low number of samples is a limitation of this study. Nevertheless, our results obtained by analyzing a higher number of samples (102 vs 16) support the no detection of SARS-CoV-2 RNA in environmental samples from hospital areas where patients with COVID-19 are attended. A possible limitation of our study is that the protocol cleaning, which was performed twice per day (morning and afternoon), influenced in the results. To avoid this limitation and given that SARS-CoV-2 can remain viable on surfaces between 4 and 72 h [10], samples were collected at different morning/afternoon hours within 15 days. Thus, the time from cleaning to sampling was variable. In any case, a possible explanation of the lack of environmental contamination could be a strict cleaning protocol with sodium hypochlorite, a high air change rate, and a negative pressure in the ICU. These facts together with the protection measures used could also explain the absence of contagion among healthcare professionals in our ICU.

## Conclusions

To our knowledge, this is the first study on environmental contamination by SARS-CoV-2 in a Spanish ICU. Our results indicate the lack of SARS-CoV-2 on the ICU surfaces supporting that personal protection, decontamination procedures, and negative pressure settings are effective in preventing environmental contamination and protecting the staff and patients inside intensive care units. The COVID-19 pandemic is a huge challenge to our health systems. The safety of professionals must be a priority to prevent the collapse of health systems and avoid transmission from hospitals to the rest of the community. The study of the transmission routes, including the role of contaminated environmental samples, is a key element to establish public health policies. Strategies to monitor surfaces and lowering the environmental viral load are necessary to minimize the risk of transmission and to maintain Ernest Shackleton’s spirit, the protection of the crew, the health professionals.

## Data Availability

All datasets on which the conclusions of this manuscript rely are presented in the main paper.
